# Bone augmentation after ectopic implantation of a cell-free collagen-hydroxyapatite scaffold in the mouse

**DOI:** 10.1038/srep36399

**Published:** 2016-11-08

**Authors:** Giovanna Calabrese, Raffaella Giuffrida, Stefano Forte, Lucia Salvatorelli, Claudia Fabbi, Elisa Figallo, Massimo Gulisano, Rosalba Parenti, Gaetano Magro, Cristina Colarossi, Lorenzo Memeo, Rosario Gulino

**Affiliations:** 1 IOM Ricerca, Viagrande, Italy; 2 Department of Biomedical and Biotechnological Sciences, Physiology Section, University of Catania, Catania, Italy; 3 Department of Medical and Surgical Sciences and Advanced Technologies, G.F. Ingrassia, “Policlinico Vittorio Emanuele”, Anatomic Pathology Section, University of Catania, Catania, Italy; 4 Finceramica Faenza, Faenza, Italy; 5 Department of Experimental Oncology, Mediterranean Institute of Oncology, Viagrande, Italy

## Abstract

The bone grafting is the classical way to treat large bone defects. Among the available techniques, autologous bone grafting is still the most used but, however, it can cause complications such as infection and donor site morbidity. Alternative and innovative methods rely on the development of biomaterials mimicking the structure and properties of natural bone. In this study, we characterized a cell-free scaffold, which was subcutaneously implanted in mice and then analyzed both *in vivo* and *ex vivo* after 1, 2, 4, 8 and 16 weeks, respectively. Two types of biomaterials, made of either collagen alone or collagen plus magnesium-enriched hydroxyapatite have been used. The results indicate that bone augmentation and angiogenesis could spontaneously occur into the biomaterial, probably by the recruitment of host cells, and that the composition of the scaffolds is crucial. In particular, the biomaterial more closely mimicking the native bone drives the process of bone augmentation more efficiently. Gene expression analysis and immunohistochemistry demonstrate the expression of typical markers of osteogenesis by the host cells populating the scaffold. Our data suggest that this biomaterial could represent a promising tool for the reconstruction of large bone defects, without using exogenous living cells or growth factors.

Repairing large bone defects frequently requires transplantation strategies to restore the anatomical and functional status of the injury sites unable to heal spontaneously.

De-vitalized allografts from cadaveric sources have been used, but they have shown a low osteogenic potential and significant risk of infections^1^,^2^. Autologous bone grafts have been considered the gold standard, due to the higher osteogenic potential and the absence of immune response. Unfortunately, bone harvesting can cause significant donor site morbidity including pain and infection^3^,^4^. Bone tissue engineering represents a promising alternative. In particular, during the past decade, the importance of tissue engineering to address limitations in tissue grafting has increased for a wide variety of diseases, including bone pathologies^5^,^6^. The field of bone tissue engineering relies on the development of biomaterials able to give the advantages of autografts without the related donor site morbidity. Scaffolds should be capable of mimicking native bone structure in terms of both mechanical and osteoinductive properties^7^,^8^,^9^,^10^,^11^,^12^,^13^,^14^. Moreover, angiogenic capability is a fundamental feature in order to improve the clinical success of bone repair^15^. The osteogenic and angiogenic capacity of the scaffold depends on its material composition, porosity and the ability to incorporate cells^6^,^15^,^16^,^17^,^18^. A large number of synthetic polymers or natural biomaterials have been used so far in regenerative medicine^19^. Among these, collagen-based biomaterials are the most highly investigated material for bone regeneration^19^,^20^, given their biomimetic properties. In order to improve the capacity of the bone substitute to recapitulate the chemical, physical and structural properties of the native young human bone and provide cells with the right osteogenic niche, the collagen I matrix has been combined with Magnesium-enriched hydroxyapatite (MHA)^10^,^12^,^14^. The osteogenic capability of this material has already been analysed *in vitro*^21^,^22^ and its ability to produce bone augmentation after ectopic implantation in the animal has been observed^21^, but a comprehensive *in vivo*, histological and molecular characterization of the bone augmentation into the implanted biomaterial, without the use of growth factors or exogenous cells, requires further investigation.

Here, in order to evaluate the osteoinductive and angiogenic potential of a cell-free collagen-MHA scaffold, in relation to its chemical composition and structural properties, we developed a bilayer scaffold made of collagen I alone (layer 1: Coll) and collagen-MHA (layer 2: Coll-MHA). The material was implanted subcutaneously into the mice. Then, osteo- and angiogenesis were analyzed both *in vivo* by Fluorescent Molecular Tomography (FMT), and *ex vivo* by histological analysis. Moreover, the population of host cells invading the biomaterial has been characterized by immunohistochemistry (IHC), as well as by quantitative real-time PCR.

## Results

### Scaffold characterization

**Figure 1:**
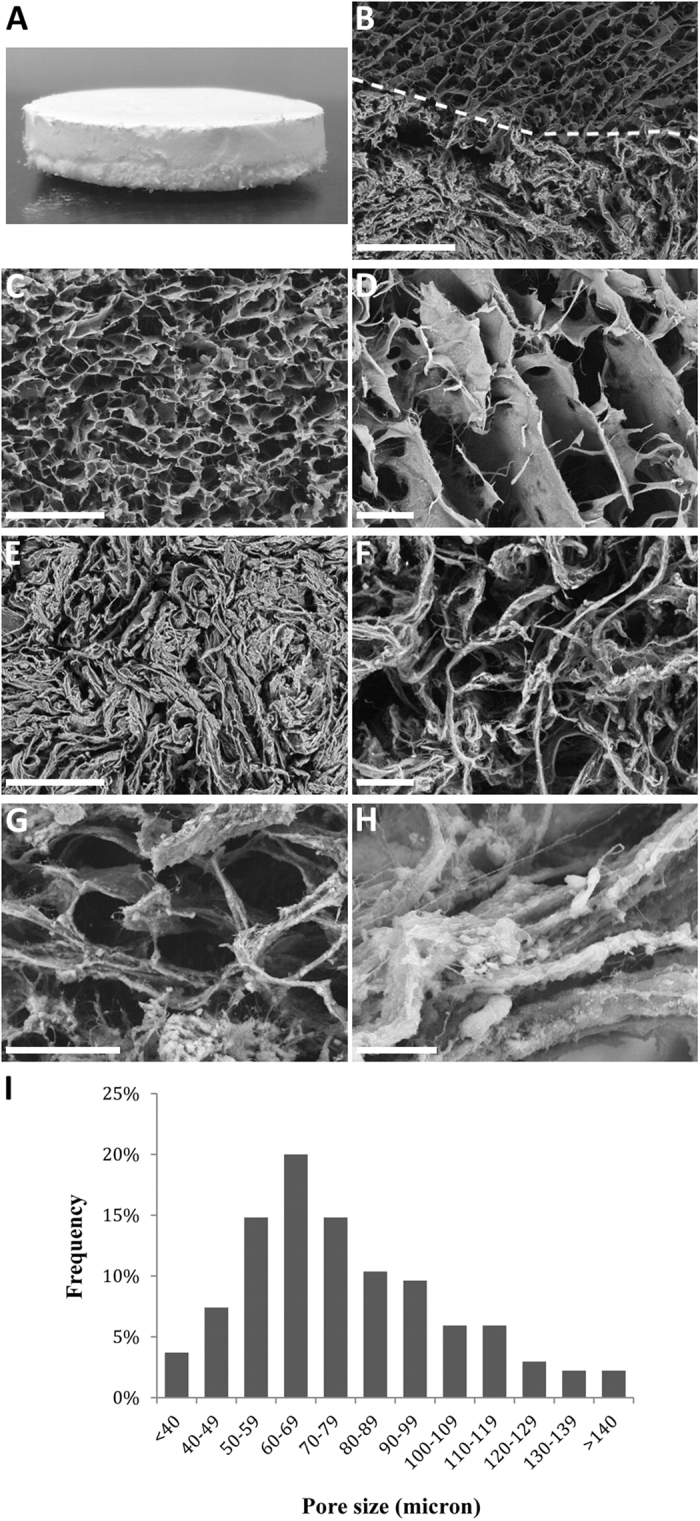
SEM imaging and analysis of the scaffold internal structure. (**A**) A photograph of the scaffold showing the external appearance and the bilayered structure; (**B**–**H**) Representative SEM images showing the interface between scaffold layers (**B**), as well as the structure of the Coll (**C**,**D**) and Coll-MHA (**E–H**) layers. At higher magnification, an open and interconnected porosity is visible within the scaffold bony layer (**G**), and MHA particles are visible within and on the surface of the collagen micro and nanosize fibers (**H**). (**I**) Analysis of the pore size distribution within the bony layer. Magnification: 50X in (**B,C,E**); 150X in (**D,F**); 300X in (**G**); 1000X in (**H**). Scale bars: 500 μm in (**B,C,E**); 100 μm in (**D,F,G**); 20 μm in (**H**).

The porosity of the biomaterial resulted 89 ± 7%. Porosity and pore size is crucial to assure cell colonization of the scaffold. Scanning electron microscopy (SEM) micrographs (Fig. 1) showed a homogeneous distribution of pores without any preferential alignment of the mineralized collagen fibers. MHA particles are visible within and on the collagen fibers (Fig. 1H). The bone substitute shows well-interconnected pores with diameters from 20 to 175 μm and a mean value of 78 μm (Fig. 1G,I). The analysis of pore size distribution has been performed, showing that about the 50% of pores have diameters ranging 50–80 μm (Fig. 1I). Large channels (300–500 μm) were sporadic and were excluded from the analysis. Scaffolds have been tested to quantify the mineralization and the collagen degradation temperature. The amount of MHA was 50 ± 1.4% of the whole structure. The collagen degradation temperature is around 319.6 ± 3.2 °C. The irritation potential was characterized by HET-CAM test. Both S and Q methods gave a score of +3 and +0.21, respectively, thus indicating that scaffolds are hypoirritating and can be safely implanted in animals.

### Evaluation of osteogenesis and angiogenesis

**Figure 2:**
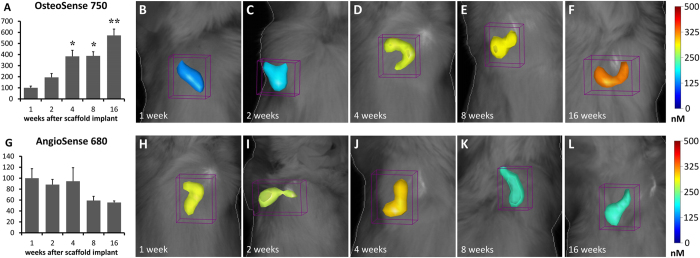
FMT images and *in vivo* quantification of osteogenesis and angiogenesis within the implanted scaffolds. The mean values calculated within groups were normalized to the mean value of 1-week group (**A,G**). The color scale indicates mean probe concentrations within the ROI. In the graph A, asterisks indicate significant differences (P < 0.05). In particular, one asterisk indicates significant difference from 1-week and 2-weeks groups while two asterisks indicate significant difference from all the other groups.

FMT imaging has shown an increasing *de novo* formation of hydroxyapatite, as revealed by the significantly increased fluorescent signal produced by OsteoSense 750 along the different time-points after implantation (Fig. 2A–F, ANOVA: F_27,4_ = 22.514; P < 0.001). In particular, a 3.8-fold increase has been found at both 4 and 8 weeks (Fig. 2A,D,E; Tukey’s post-hoc test: P < 0.05) and a 5.7-fold increase has been seen at 16 weeks (Fig. 2A,F; Tukey’s post-hoc test: P < 0.05), as compared to the 1-week group (Fig. 2A,B). Conversely, the fluorescent signal produced by AngioSense 680 appears relatively high until 4 weeks (Fig. 2G–J), and then apparently decreases by about 40%, although this decrease was not statistically significant (Fig. 2G,K,L; ANOVA: F_27,4_ = 1.801; P > 0.05).

Prior to implantation, scaffolds have a volume of 250 mm^3^. The volume of explanted scaffolds was reduced by average 20% at 1 and 2 weeks, and by average 34% at 4–16 weeks. Starting at 4 weeks after implantation, explanted scaffolds appeared white and lost their soft consistency cause the increasing mineralization.

**Figure 3:**
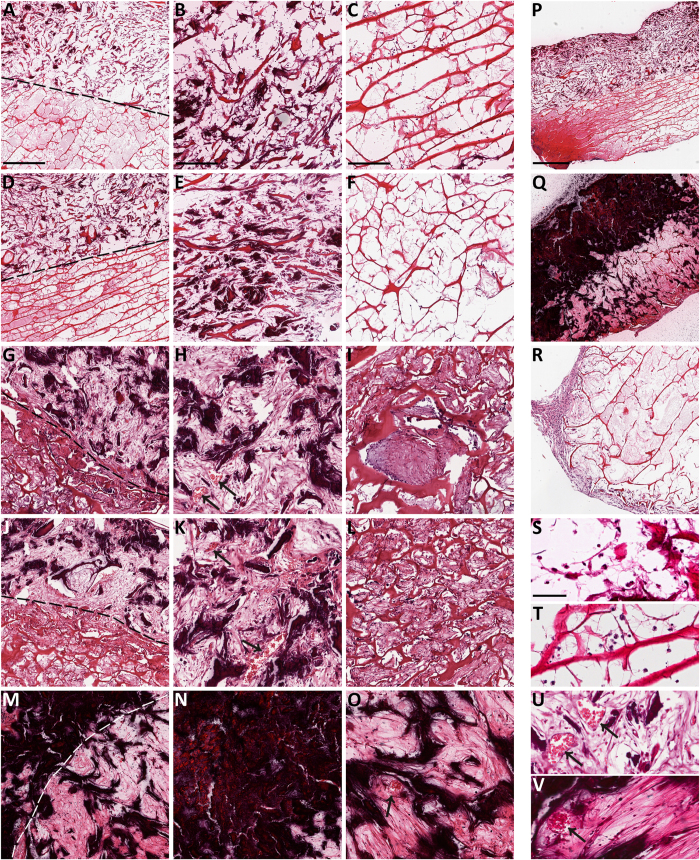
Time-course of osteogenesis and angiogenesis within the implanted scaffolds as seen by H&E at 1 week (**A–C**), 2 weeks (**D–F**), 4 weeks (**G–I**), 8 weeks (**J–L**) and 16 weeks (**M–O**). The difference between the Coll-MHA and the Coll layers is evident in the left column, showing the interface between the two layers, where the upper half is the bony layer, while the bottom half is the Coll layer. The second and third columns show the increasing mineralization of the Coll-MHA layer (from **B** to **N**) and the formation of fibrosis in the Coll layer (from **C** to **O**). A bone-like tissue is also visible in the Coll layer starting at 4–8 weeks (**I–L**), with signs of mineralization that become stronger at 16 weeks (**O**). The entire process could be visualized by comparing the whole section at 1 and 16 weeks (**P**,**Q**). Arrows in (**H,K,O**) indicate vascular structures, which are visible at higher magnification in the enlarged inserts (**U,V**). Scaffolds are wrapped by a layer of fibroblast-like cells (**R**) that progressively invade the inner material (**A–O**). At 1 week, some granulocytes were present into the material (**B**,**C**, enlarged in **S**,**T**). Scale bars in (A)for the left column: 500 μm; in (**B,C**) for the relative columns: 250 μm; in (**P**) for (**P–R**): 1 mm; in (**S**) for (**S–V**) 50 μm.

Hematoxylin and eosin (H&E) staining reveals that the biomaterial undergone a dramatic change during the post-grafting period (Fig. 3). As early as the first week (Fig. 3A,B), a visible mineralization of the bony layer (Coll-MHA) started and constantly increased overtime, as visualized by the formation of dark areas within the tissue. At 16 weeks post-implantation, the bony layer appeared completely mineralized (Fig. 3N, see also Fig. 3P,Q for a comparison between the 1 week and 16 week time-points). The biomaterial was wrapped by a layer of host cells (mainly fibroblasts) starting at 1 or 2 weeks (Fig. 3R), which then gradually invaded the inner part of the material and filled the spaces between collagen fibers and mineralized trabecula of both layers. This process was particularly robust at 4 weeks (Fig. 3H,I) and subsequent time-points (Fig. 3J–L,M,O). The scaffold layer made of collagen alone (Coll) also undergone a visible modification (Fig. 3). Together with the accumulation of fibroblast-like cells, the material undergone an evident fibrosis (Fig. 3I,L,O). Interestingly, starting at 4 weeks post-implantation, the Coll layer showed a bone-like morphology (Fig. 3G,I,J,L) and a hint of mineralization appeared as dark spots within the matrix (Fig. 3I,L), which became stronger at 16 weeks post-implantation (Fig. 3M,O,Q).

Furthermore, H&E staining confirms FMT analysis regarding the vascularization of the scaffold, by showing the presence of structures clearly resembling blood vessels. Vascular structures were particularly abundant at 4 and 8 weeks post-implantation, especially within the Coll-MHA layer (Fig. 3H,K,U, arrows). At 16 weeks, vascular formations were not visible within the bony layer, whereas some blood vessels were still visible, although reduced in number, within the Coll layer (Fig. 3O,V, arrows).

The H&E staining confirms the high biocompatibility of the biomaterial, as suggested by the absence of necrotic areas and the small and transient inflammatory reaction in explanted scaffolds. In fact, the presence of granulocytes is evident at one week after implantation (Fig. 3B,C,S,T), but they were fewer at two weeks (Fig. 3E,F) and completely disappeared at the longer time-points.

**Figure 4:**
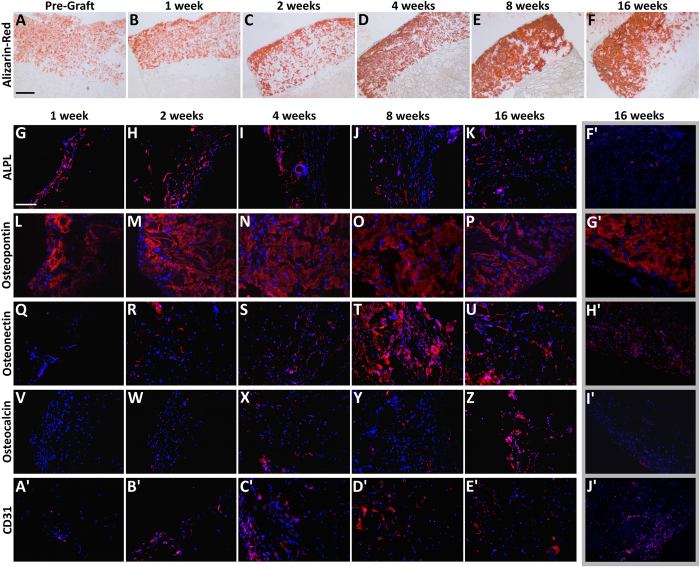
Alizarin Red S and immunofluorescence staining. (**A**–**F**) Alizarin Red S staining showing the time-course of increasing calcium mineralization within the implanted scaffolds. It is evident that the calcium deposits are mainly localized in the bony layer. (**G**–**E**′) Panel of fluorescence microscope images showing the time-course of expression of crucial osteogenic and angiogenic markers by the host cells populating the Coll-MHA layer of the scaffolds. (**F′–J′**) Examples of fluorescence microscope images showing the expression of osteogenic and angiongenic markers in the Coll layer: ALPL and osteocalcin have shown a low signal at all time-points (examples in **F′,I′**), whereas an increased expression of osteopontin (**B′**) and a little increase of osteonectin (**H′**) and CD31 (**J′**) could be observed at week 16. Scale bars: 500 μm in **A** for (**A–F**); 100 μm in (**G**) for all fluorescence images.

Alizarin Red S was used to stain calcium deposits and confirm bone augmentation. Tissue sections of pre-graft scaffolds resulted stained only in the Coll-MHA layer, since a small percentage of calcium is present in its formulation (Fig. 4A). Then, the mineralization appears gradually increasing after implantation and confined to the bony layer (Fig. 4B–F).

IHC of anti-alkaline phosphatase (ALPL), a typical marker of matrix maturation, exhibits a quite strong signal, in the Coll-MHA layer, during the first two weeks after implantation (Fig. 4G,H) that progressively decreased up to week 16 (Fig. 4I–K). Osteopontin, which is involved in bone remodeling^23^, shows a very strong signal at each time-point, with a peak at week 2, gradually decreasing up to week 16 (Fig. 4L–P). Osteonectin, a glycoprotein secreted by osteoblasts and involved in matrix mineralization, reveals a weak expression during the first four weeks (Fig. 4Q–S), followed by a marked increase at week 8 (Fig. 4T) and a partial reduction at week 16 (Fig. 4Q–U). IHC analysis of osteocalcin, a specific marker of mature osteoblasts, exhibits no signal during the first two weeks (Fig. 4V,W). Then, at week 4, a very weak signal appears, remaining almost unchanged up to week 8 (Fig. 4X,Y) and strongly increasing at week 16, (Fig. 4Z). The expression of CD31 endothelial marker (Fig. 4A′–E′) was in line with the results of H&E staining, showing a weak CD31 signal at the first week (Fig. 4A′) that gradually increased up to week 8 (Fig. 4D′). At week 16, CD31 expression appeared markedly reduced (Fig. 4E′).The IHC signal of these markers in the Coll layer was very low at all time-points, except for osteopontin (Fig. 4G′), which appeared strongly expressed at 16 weeks post-implantation, and a moderate expression of osteonectin (Fig. 4H′) and CD31 (Fig. 4J′), which were moderately expressed at 16 weeks.

**Figure 5:**
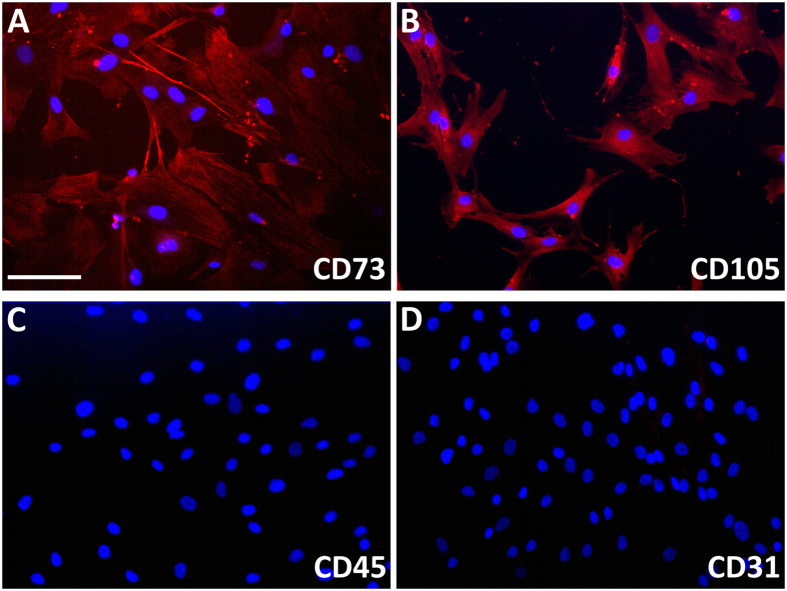
Characterization of murine cells isolated from the explanted scaffolds. IHC analysis of positive (CD73 and CD105) and negative (CD45 and CD31) MSCs markers to verify stemness of murine cells at 4 weeks after implantation. Scale bar: 100 μm.

As shown in Fig. 5, mesenchymal stem cells (MSCs) could be isolated from the scaffolds explanted at 4 weeks post-implantation. As expected, cells show a positive signal for the stem cell markers CD73 and CD105 (Fig. 5A,B), whereas the expression of CD45 and CD31 results not detectable (Fig. 5C,D). MSCs, with similar phenotype, have also been isolated from scaffolds at 2, 8 and 16 weeks (not shown), although the isolation from scaffolds at 8–16 weeks post-implantation was difficult, probably because of the high rate of mineralization and fibrosis.

### Expression of genes linked to osteogenesis

**Figure 6:**
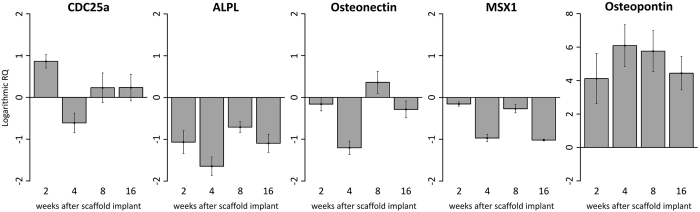
RT-qPCR analysis. Plots of logarithmic RQ values showing the time-course of the expression of genes involved in cell proliferation and osteogenesis by host cells populating the implanted scaffolds.

RT-qPCR has been performed on total RNA extracted from explanted scaffolds in order to quantitatively evaluate the expression of genes involved in osteogenesis and cell proliferation at the mRNA level. The expression of target genes at 2, 4, 8 and 16 weeks has been compared to mRNA levels at week 1. Logarithmic RQ values are reported in Fig. 6. Cell division cycle 25A (CDC25A) shows an increase of almost one order of magnitude during the second week with a subsequent decrease and reduced modulation until week 16. ALPL and msh homeobox 1 (MSX1) show reduced expression during weeks 2–16 with peaks of negative regulation during the 4th and 8th week. Osteonectin shows a distinctive peak of expression at week 8 with about half order of magnitude in mRNA increase. Osteopontin mRNA concentration increases during the whole period with a massive modulation up to 6 order of positive modulation during the 4th week.

## Discussion

The regeneration of large bone defects remains a big challenge in orthopedics and an ideal solution is still lacking. The development of new biomaterials able to restore the bone structure and functional properties represents a promising approach. The potential of novel collagen/hydroxyapatite biomimetic scaffolds has been previously analyzed to evaluate the physicochemical properties^12^,^14^, as well as the capability of inducing bone formation in combination with human MSCs *in vitro* and *in vivo*^12^,^14^,^22^. In the present study, we have investigated the ability of this biomaterial of inducing ectopic bone formation *in vivo*, after transplantation in the mouse as a cell-free scaffold. To our knowledge, this is the first study where osteogenesis and angiogenesis within the scaffold have been evaluated *in vivo* in a time-course manner, by using a FMT technology. Moreover, we have evaluated *ex vivo* the role of the scaffold composition, in terms of MHA content, in the recruitment of host cells and mineralization of the synthetic bone matrix. Finally, osteogenesis has been deeply characterized by analyzing the time-course expression of several genes involved in these processes.

Suitability of biomimetic materials requires their complete biocompatibility in order to avoid rejection and chronic inflammation. Biocompatibility can be defined as the ability to support host cell proliferation and differentiation, thus promoting the formation of the extracellular matrix, without any toxic or injurious effect. Moreover, osteogenesis should be paralleled by the creation of new blood vessels, which are important to support the formation of new bone tissue.

We have recently demonstrated that Coll-MHA scaffolds are able to commit human MSCs towards osteogenic differentiation *in vitro*^22^. Here, the ability of this material to recruit host cells and promote ectopic bone augmentation has been assessed *in vivo*. To this aim, the scaffolds have been subcutaneously implanted in the mice dorsum and novel bone formation and angiogenesis have been measured at different time-points by FMT. All animals remained healthy for the entire period of the study, showing no noticeable sign of toxicity or other adverse effects. Moreover, examined samples present little or no evidence of inflammatory events, thus confirming the high biocompatibility of the implants. *In vivo* results demonstrate the mineralization of the biomaterial, as indicated by the increasing hydroxyapatite production *in vivo*. Moreover, *ex vivo* characterization of the ossified matrix clearly indicate that scaffolds are rapidly populated by the host cells and strongly mineralized. Immunohistochemistry and gene expression analysis have also shown that these cells may express osteogenic markers. Similar results by Minardi and colleagues^21^ have demonstrated that a Coll-MHA scaffold could be spontaneously mineralized *in vivo.* Interestingly, we noticed that the *in vivo* mineralization was prominent within the bony layer of the scaffold but, although the main process occurring in the collagen layer was fibrosis, a bone-like morphology, with an evident though limited mineralization was also observed in this material at 8–16 weeks after implantation. The differential properties of Coll and Coll-MHA biomaterials in inducing osteogenic differentiation of exogenous MSCs has already been shown both *in vitro*^12^,^22^ and *in vivo*^14^, thus demonstrating that these differences could depend from both scaffold composition and culture conditions of cells. Here, by grafting a bilayer scaffold without any trophic or cellular supplement, we have demonstrated that the ability of inducing migration and differentiation of resident cells is an intrinsic property of the biomaterial, and the observed difference in the subsequent changes of the material properties should be linked to the MHA content. These results are in line with data reported in literature^21^,^24^,^25^. Collagen, which is the main protein component of bone, is able to induce MSCs differentiation into osteoblasts^24^. Moreover, it has been shown that biomaterials containing hydroxyapatite could improve cell proliferation and differentiation, and promote the production of bone matrix more efficiently than observed for the scaffold without hydroxyapatite^25^.

As shown by histology, new vascular structures appeared into the implanted scaffolds after implantation. This process has also been shown *in vivo* by FMT analysis and confirmed by the expression of CD31. The formation of well-organized blood vessels within both layers of the biomaterial is an important finding because it indicates a good graft-host interaction and represents an important condition for the long-term cell survival. However, in the present experiments, after an initial increase, the number of blood vessels seems reduced again in the mineralized matrix, probably because of the increasing formation of new mineralized tissue. To overcome this problem, the collagen-MHA material should include small areas of collagen without MHA, where vascular structures could still develop, as shown here in the collagen layer, thus improving the vascularization of the bony layer^26^,^27^,^28^.

Along with the scaffold composition, the local tissue microenvironment is a key regulator of the cell behavior, so further studies are needed to address this issue by grafting the scaffolds into other organs, of course including lesioned bone sites. The orthotopic implantation of this material should result in a different organization of the newly formed mineralized tissue.

*Ex vivo* molecular characterization of explanted samples confirms that host cells populating the scaffolds include a subpopulation of MSCs. These cells were probably committed through the well-known molecular pathways^29^,^30^,^31^,^32^,^33^,^34^ that involve the expression of early markers, such as ALPL and osteopontin during the first two weeks, followed by an increase in the expression of late markers, such as osteocalcin and osteonectin. Accordingly, the expression of genes involved in cellular proliferation and cell cycle progression, namely CDC25a is greatly increased during the initial stages of differentiation. The transcript shows an expression peak during the second week and rapidly decreases in concentration as the differentiation process continues. The osteogenic commitment of host cells invading the biomaterial could be responsible, at least in part, for the strong formation of calcium deposits shown in the bony layer, and this assumption is confirmed by the evidence that MSCs express osteopontin and produce mineralized material after osteogenic commitment *in vitro*^35^.

In conclusion, our findings demonstrate that a collagen-hydroxyapatite scaffold made by a biologically inspired method and ectopically implanted into the mice is capable, by itself, to recruit host cells that invade the scaffold and promote bone augmentation, probably as a result of osteogenic commitment. Moreover, vascular structures appeared spontaneously within the biomaterial, thus representing a promising result for a successful bone formation and integration to the host. Given the demonstrated safety of this biomaterial, the use of scaffolds alone, without additional use of growth factors or *in vitro* manipulated cells could represent a safe and promising approach for bone healing, as confirmed by similar studies carried out with the use of similar or different materials^21^,^36^. Further studies are needed, however, to better characterize the scaffold properties in different tissues environments, including lesioned bone.

## Methods

### Scaffold preparation and characterization

The biomimetic scaffold used in this study (Fin-Ceramica Faenza SpA, Faenza, Italy) has a cylindrical shape, with an 8 mm diameter and 5 mm high, and a bilayered structure (Fig. 1A,B). In particular, one layer (3 mm depth) consists of equine type I collagen (Coll), whereas the other layer (2 mm depth) is made of a mineralized blend of type I collagen (30%) and Mg-HA (70%), mimicking a bone-like tissue^12^,^14^ (Coll-MHA). The scaffold was developed through a bio-inspired process, as previously described^22^. The present study was designed to verify the scaffold capability of driving bone augmentation. For this reason, the scaffold characterization focused on the assessment of porosity, mineral content and potential irritancy. Porosity and mineral content have been assessed respectively by SEM and thermogravimetric analysis as previously described in more detail^22^. The irritancy potential has been evaluated by HET-CAM test, a sensitive test recently proposed to evaluate biocompatibility before animal testing^37^,^38^. The test was performed by direct contact (S method), as well as by putting the extract (Q method) over the chorioallantoic membrane (CAM) of fertilized chicken eggs on incubation day 9. Subsequently, the eggs were incubated for 7 days and then inspected. Any modification on the vascular system and on albumin, such as bleeding, lysis and clot were scored to quantify the intensity of irritating phenomena at the CAM level. The material is defined hypoirritating with S score under 6 and Q score under 0.8.

### Animals and experimental design

Female mice (n = 52) (BALB/cOlaHsd, 6 weeks aged, weight: 17–22 g; Harlan Laboratories) were used. Animal handling was carried out in accordance with the EU Directive 2010/63/EU and all experiments have been approved by the Italian Ministry of Health. Animals were housed in groups of four in individually ventilated cages, with *ad libitum* access to water and food, with standard conditions of temperature (22 ± 2 °C) and relative humidity (50 ± 5%) and a light/dark cycle of 12/12 h. Surgical procedures were executed under aseptic conditions, keeping mice under gas anesthesia (isoflurane). All efforts were made to minimize the number of animals used and their suffering. Scaffolds were implanted into a subcutaneous pocket in the dorsum of the animals. The implanted mice were randomly assigned to five groups: 1 wk (n = 8), 2 wk (n = 8), 4 wk (n = 12), 8 wk (n = 8), 16 wk (n = 12), and they were sacrificed by intracardiac injection of Tanax (MSD Animal Health Srl) under deep anesthesia at 1, 2, 4, 8 or 16 weeks after surgery, respectively. The scaffolds were removed and collected for analysis. Four additional animals without scaffold implantation were used as negative controls for *in vivo* imaging.

### *In vivo* FMT

In order to functionally assess the time-course of osteogenesis and angiogenesis within the implanted scaffolds, animals were analyzed by FMT (FMT 2500, Perkin Elmer). In particular, the implanted animals as well as four intact mice, received an injection of 100 μl of OsteoSense 750EX plus 100 μl of AngioSense 680EX (Perkin Elmer) into the tail vein, according to the manufacturer’s instructions. Twenty-four hours later, FMT scans were performed. During the acquisition, animals were kept under isoflurane anesthesia. Acquisition and data analysis were carried out using the TrueQuant software (Perkin Elmer). For analysis, the region of interest (ROI) was selected and the extent of osteogenesis or angiogenesis was quantified by measuring the amount of fluorescence probe (in pmol) into the ROI after choosing a concentration threshold. This threshold has been determined by keeping the volume of ROI constant (50 mm^3^). The animals were sacrificed immediately after imaging.

### Isolation and characterization of murine cells from the scaffold

Some scaffolds explanted at 4 weeks (n = 2) after implantation were used. They were reduced in small pieces and digested at 37 °C for 2 h in PBS containing collagenase type I (0.5 U/ml). The suspension was filtered, suspended in mesenchymal stem cells growth medium (Lonza Group Ltd.) and plated on 8-well chamber slides (Corning) at a density of 5000 cells/cm^2^. The medium was changed twice/week until reaching 70–80% confluence. Then, cells were fixed with 4% paraformaldehyde and stained by using the following antibodies: mouse anti-CD73 (1:25, Novus Biologicals); rabbit anti-CD105 (1:50, Novus Biological), rabbit anti-CD31 (1:50, Novus Biological) and rabbit anti-CD45 (1:100, Epitomics). Briefly, slides were blocked for 1 h at room temperature (RT) with 5% normal donkey serum and 0.3% Triton-X100 in PBS and incubated for 2 h, at RT with the primary antibodies. Then, sections were processed for the secondary incubation with the appropriate Alexa Fluor 568 antibody (Life Technologies) at the dilution of 1:2000. After washing, slides were counterstained with DAPI (1:10 000), mounted and examined by using a Leica DMI 4000B fluorescence microscope (Leica Microsystems).

### Histology

Two scaffolds for each time-point were used for IHC. Scaffolds were explanted and fixed for 2 h in 4% paraformaldehyde, embedded in paraffin and cut in 3.0 μm-thick sections. For staining, sections were blocked with 0.4% Triton-X100 and 4% bovine serum albumin and then incubated overnight at 4 °C with one of the following rabbit polyclonal antibodies: anti-osteopontin (1:250, Novus Biological), anti-osteocalcin (1:50, LSBio), anti-alkaline phosphatase (ALPL; 1:50, LSBio), anti-osteonectin (1:50, LSBio) and anti-CD31 (1:50, Novus Biological). Then, sections were incubated for 1 h at RT with Alexa Fluor anti-rabbit 568 (1:2000, Life Technologies Italia). Afterwards, slides were counterstained with DAPI (1:10 000) and mounted with Permafluor (Thermo). The immunostaining specificity was verified by omitting the primary antibody. Alternate sections were stained with H&E or Alizarin Red S. Alizarin Red S staining was performed as previously reported^22^,^37^. The stained slides were examined by using a Leica DMI 4000B fluorescence microscope.

### Quantitative Real Time Polymerase Chain Reaction (Q-PCR)

Total RNA was extracted from scaffolds (5 scaffolds/time-point) by using TRIzol reagent (Invitrogen Inc.). Reverse transcription of total RNA was performed using High-Capacity cDNA Reverse Transcription Kit (Applied Biosystems). The expression levels of genes involved in osteogenesis and cell cycle regulation, including ALPL, CDC25A, osteonectin, osteopontin and MSX1 were measured using SYBR Green method on a 7900HT Real Time PCR (Applied Biosystems). Specific primers for selected murine mRNA were designed using primer blast^39^ using exon-exon junctions on messengers as target region (see Table 1). The mRNA level of glyceraldehyde-3-phosphate dehydrogenase (GAPDH) and beta-tubulin (Tubb4a) were used as endogenous controls.

### Statistical analysis

Data were analyzed either as raw data or as mean ± s.e.m., as appropriate. Differences between experimental groups were evaluated by using one-way ANOVA followed by Tukey’s *post hoc* test. For all experiments, a P value of <0.05 was considered significant. All analyses were performed by means of Systat Software.

## Additional Information

**How to cite this article**: Calabrese, G. *et al*. Bone augmentation after ectopic implantation of a cell-free collagen-hydroxyapatite scaffold in the mouse. *Sci. Rep.* **6**, 36399; doi: 10.1038/srep36399 (2016).

**Publisher’s note:** Springer Nature remains neutral with regard to jurisdictional claims in published maps and institutional affiliations.

## Figures and Tables

**Table 1:** Primer sequences.

Target gene	Forward	Reverse
CDC25A	GGCAAGCGTGTCATTGTTGT	GGCTCACAGTAAGACTGGCA
ALPL	GACCCTTGACCCCCACAAT	CGCCTCGTACTGCATGTCCCCT
Osteonectin	TAGCACACAGCCTACCACAAG	AGCAACTTCAGTCTGCTGAGGG
MSX1	CCACTCGGTGTCAAAGTGGA	GAAGGGGACACTTTGGGCTT
Osteopontin	AGTTTCGCAGACCTGACATCCAGT	TTCATAACTGTCCTTCCCACGGCT
TUBB4a	GACGTGAGTACTGCTCCGC	CTTGCAGGTGCACGATTTCC
GAPDH	TGTGAACGGATTTGGCCGTA	ACTGTGCCGTTGAATTTGCC
